# Comprehensive analysis of element and metabolite content between the seeds of *Apocynum venetum* and *Apocynum pictum* provides new sights for the salt tolerance in *Apocynum*

**DOI:** 10.3389/fpls.2025.1611975

**Published:** 2025-06-25

**Authors:** Li Jiang, Yu Zhao, Zijun Jiang, Yukun Wu, Bin Wen, Siyu Zhang, Junyi Zhan, Nana Su

**Affiliations:** ^1^ State Key Laboratory of Desert and Oasis Ecology, Xinjiang Institute of Ecology and Geography, Chinese Academy of Sciences, Urumqi, China; ^2^ College of Life Sciences, Nanjing Agricultural University, Nanjing, China

**Keywords:** *Apocynum venetum*, *Apocynum pictum*, seed, salt tolerance, element, metabolomic

## Abstract

The genus *Apocynum* (family *Apocynaceae*), which includes perennial herbaceous plants, demonstrates remarkable stress tolerance, particularly in saline environments. In this study, we investigated interspecific variations in salt tolerance during the critical germination stage between two predominant *Apocynum* species, *A. venetum* and *A. pictum*. Since seed metabolic processes play a pivotal role in determining germination efficiency and subsequent plant development, seed biochemistry has become an essential parameter for evaluating seed viability and stress resilience. To elucidate the superior salt tolerance mechanism of *A. pictum* seeds compared to *A. venetum* seeds, we conducted quantitative analyses of mineral elements using inductively coupled plasma-mass spectrometry (ICP-MS) combined with untargeted metabolomics profiling. The results revealed that most mineral element concentrations were higher in *A. pictum* seeds than in *A. venetum* seeds. Furthermore, metabolomic characterization highlighted significant interspecific divergence in both primary and secondary metabolites, especially flavonoids. These multi-omics findings indicate that the coordinated accumulation of stress-protective elements and antioxidant metabolites is a critical determinant of enhanced salt tolerance in *A. pictum* seeds. Our study provides novel insights into the molecular mechanisms underlying halophytic adaptation in *Apocynum* species, laying a theoretical foundation for future functional genomics research and genetic engineering strategies aimed at improving crop salt tolerance.

## 1 Introduction

*Apocynum*, a perennial herbaceous plant or small shrub species complex, demonstrates remarkable ecological adaptability across northern China’s arid, semi-arid, and saline-alkaline regions, including extreme desert environments in the northwest (Jiang et al., 2021). This xerophytic genus has evolved specialized physiological mechanisms to withstand drought and salinity stresses, though the underlying molecular regulatory networks remain poorly characterized. The growing research interest in *Apocynum* stems from its multifunctional applications as a sustainable industrial crop for high-quality fiber production, a medicinal resource enriched with bioactive secondary metabolites, and an ecological pioneer species for vegetation restoration in marginal lands (Thevs et al., 2012). Despite these recognized values, current investigations disproportionately focus on phytochemical properties and fiber utilization, leaving critical knowledge gaps in understanding the genetic determinants of stress tolerance and nutrient acquisition strategies under environmentally constrained conditions (Xiang et al., 2023).

Drought and salinity stress constitute pivotal abiotic constraints that profoundly influence plant biogeographical patterns, compromise agricultural productivity, and jeopardize global food security. As principal environmental stressors, drought elicits multiscale physiological disruptions, notably impeding cellular proliferation through suppression of cyclin-dependent kinase (CDK) activity, particularly during critical developmental phases such as seedling establishment. These metabolic perturbations manifest morphologically as reduced leaf expansion rates, diminished total leaf area, and accelerated senescence of mature foliage—key xeromorphic adaptations that mitigate water loss through stomatal closure and preferential abscission of older leaves (Fang and Xiong, 2015). Concurrently, such water conservation strategies incur significant photosynthetic penalties, including degradation of chlorophyll pigments and carotenoid depletion, ultimately impairing light-harvesting efficiency (Yao et al., 2018). At the cellular level, dehydration-induced membrane disorganization and cytoplasmic viscosity elevation provoke macromolecular destabilization, characterized by aberrant protein-protein interactions and subsequent aggregation phenomena. These structural compromises are exacerbated by osmolytes concentration effects and altered membrane fluidity under prolonged water deficit conditions.

Salt stress constitutes a major abiotic stressor that significantly impedes growth and yield formation in nearly all major crops. Notably, Na+ exerts more pronounced phytotoxic effects compared to other ions under saline conditions (Chen et al., 2007). The multifaceted impacts of salt stress span critical physiological processes including photosynthetic efficiency, respiratory metabolism, and developmental regulation, while concurrently disrupting ionic homeostasis, enzymatic activities, and phytohormonal balance (Parihar et al., 2015). During initial salt exposure, plants exhibit suppressed leaf expansion rates. Progressive salinity intensification leads to significant reductions in leaf area index, accompanied by decreased leaf water potential, relative water content, and transpiration rates. Concurrently, biomass allocation patterns are altered, manifesting as diminished fresh and dry weights in both aerial (stem) and subterranean (root) tissues. At cellular level, osmotic stress-induced stomatal closure combines with ionic toxicity to induce chloroplast ultrastructural damage, characterized by thylakoid membrane dilation, grana disintegration, and abnormal starch granule accumulation. Furthermore, salt-triggered denaturation of photosynthetic enzymes compromises light reaction efficiency and Calvin cycle functionality, thereby diminishing carbon assimilation capacity. While plants initially upregulate respiratory activity to meet energy demands for stress adaptation, sustained salinity exposure ultimately suppresses respiratory intensity in a time- and concentration-dependent manner (Kumar et al., 2021).

To adapt to environmental changes, plants have evolved a range of defense mechanisms to withstand various stressors, including drought and salinity. Research has demonstrated that under conditions of drought and salt stress, the activity of key plant antioxidant enzymes—such as catalase (CAT), peroxidase (POD), and superoxide dismutase (SOD)—increases significantly. Additionally, the levels of osmoregulatory substances like proline, glutamic acid, and soluble sugars also rise (Liu et al., 2014). The accumulation of these solutes effectively reduces the osmotic potential within cells to some extent, thereby enhancing the plant’s resilience against drought conditions. Furthermore, the concentration of secondary metabolites in plants varies with the intensity of drought stress; for instance, there is an observed increase in total flavonoid content in wheat leaves and elevated levels of phenolic compounds in poplar trees.

Seed germination and seedling growth represent the most sensitive stages to salt and drought stress, making them critical phases for investigating plant stress resistance (Chen et al., 2022). The adaptability of seeds to adverse conditions directly influences their germination, which subsequently affects population renewal, size, and distribution range. Both salt stress and drought stress can induce detrimental physiological and biochemical changes in germinating seeds (Jiang et al., 2021). These stresses can impact seed germination through mechanisms such as osmotic pressure, ion-specific effects, and oxidative stress. Furthermore, the diverse substances present within seeds can significantly influence the germination rate under varying conditions as well as the subsequent growth and development of seedlings in response to both drought and salt stresses.

Research indicates that the two primary varieties of *A pocynum*, *A. pictum* and *A. venetum*, exhibit differences in salt and drought resistance among their seedlings and mature plants (Peng et al., 2012). To investigate whether these differences in resilience to salt stress and drought stress manifest during the seed stage, we employed ICP-MS to measure various elements present in the seeds of both varieties. Additionally, we conducted a comprehensive analysis of the types and concentrations of metabolites using widely targeted metabolomics. Through this approach, we aim to further elucidate the potential mechanisms underlying *Apocynum* tolerance to adverse conditions and environmental stresses.

## 2 Materials and methods

### 2.1 Acquisition of *A. pictum* and *A. venetum* seeds

Seeds of *A. pictum* and *A. venetum* were collected in Yuli, Xinjiang, China (coordinates N85.54443, E41.30025). Then, the mature healthy seeds are dried and stored at room temperature for further usage.

### 2.2 Seed germination and stress treatment

After disinfection with ethanol and sodium hypochlorite, the seeds were thoroughly rinsed multiple times with sterile double-distilled water. The sterilized seeds were then carefully picked up one by one using tweezers and placed onto 1/2 MS medium for germination experiments. To simulate salt stress treatment, varying concentrations of NaCl solution were incorporated into the 1/2 MS medium to achieve final NaCl concentrations of 0 mM, 50 mM, 100 mM, 200 mM, 400 mM, and 600 mM. Similarly, to induce drought stress treatment, different concentrations of polyethylene glycol (PEG6000) solution were added to the culture medium so that the final concentration of PEG6000 reached levels of 0%, 5%, 10%, 15%, 20%, 25%, 30%, and 40%. According to the Michael and Kaufmann’s empirical formula, the osmotic potentials corresponding to the above PEG concentrations at 25 °C are approximately 0, −0.06, −0.17, −0.32, −0.53, −0.79, −1.10, −1.88MPa. Following the placement of seeds on various solid media containing different treatments, they were incubated in a dark environment at a temperature of (25 ± 2 °C) for germination.

### 2.3 Microwave digestion of seeds and elemental determination using ICP-MS

The seeds were washed and dried in a 65 °C oven until they reach a constant weight. 2g well-ground seed powder using a mortar were weighed to be tested. 0.1g powder were accurately weighed and then thoroughly digested using nitric acid on the microwave digestion apparatus. After digestion, the obtained liquid was diluted with deionized water and performed subsequent ICP-MS measurements. The specific ICP-MS method refers to Zhu’s (Zhu, 2023).

### 2.4 Extraction of metabolites from seeds

Using vacuum freeze-drying technology, the samples were placed in a freeze dryer (Scientz 100F), and then ground into powder use a grinder (MM 400, Retsch; 30 Hz, 1.5 minutes). Next, an electronic balance (MS105D μ) was used to weigh 50 mg of sample powder and 1200 μL of a 70% methanol internal standard aqueous solution extract pre-cooled at -20°C was added. The sample was vortexed once every 30 minutes for 30 seconds, a total of 6 times. After centrifugation (at a speed of 12000 rpm for 3 minutes), the supernatant was aspirated and the sample was filtered through a microporous membrane (with a pore size of 0.22 μm) and stored in an injection bottle for UPLC-MS/MS analysis.

### 2.5 UPLC and ESI-Q TRAP-MS/MS

The sample extracts were analyzed using an ultra-performance liquid chromatography-tandem mass spectrometry (UPLC-MS/MS) system coupled with electrospray ionization (ESI) and Tandem mass spectrometry system. The analysis conditions are as follows: UPLC: Chromatographic column, Agilent SB-C18 (1.8 μm, 2.1 mm * 100 mm); The mobile phase consists of solvent A (pure water containing 0.1% formic acid) and solvent B (acetonitrile containing 0.1% formic acid). A gradient program was used with initial conditions of 95% A and 5% B for sample measurement. Program the linear gradient of 5% A and 95% B within 9 minutes, and maintain the composition of 5% A and 95% B for 1 minute. Subsequently, adjust the composition of 95% A and 5.0% B within 1.1 minutes and maintain it for 2.9 minutes. Set the flow rate to 0.35 mL/min; Set the column temperature box to 40°C; The injection volume is 2 μL. Connect the effluent alternately to ESI triple quadrupole linear ion trap (QTRAP) MS.

The operating parameters of ESI source are as follows: source temperature of 500°C; Ion spraying voltage (IS) 5500 V (positive ion mode)/-4500 V (negative ion mode); Ion source gas I (GSI), gas II (GSII), and curtain gas (CUR) are set to 50, 60, and 25 psi, respectively; Collision activated dissociation (CAD) is relatively high. QQQ scanning was obtained through MRM experiments, with the collision gas (nitrogen) set to medium. The DP (declustering potential) and CE (collisionenergy) used for single MRM conversion are further optimized through DP and CE. Monitor a specific set of multiple reaction monitoring (MRM) channels for each period based on the metabolites eluted during this period.

### 2.6 Quality control

Principal component analysis (PCA) was applied to analyze the differences in metabolites in seeds of two different varieties of *Apocynum*. This analysis method is commonly used to study how to reveal the internal structure between multiple variables through a few principal components, that is, to derive a few principal components from the original variables, so that they retain as much information as possible about the original variables and are not correlated with each other. The usual mathematical processing is to linearly combine the original multiple indicators as a new comprehensive indicator PCA chart to analyze sample distribution, in order to determine the trends, anomalies, and variability within and between groups based on the original data. The cohesion of quality control (QC) samples is a sign of data repeatability and equipment stability. Better instrument stability and higher consistency of collected data are reflected in tighter QC sample grouping.

### 2.7 Data analysis

PCA was performed on grouped samples for comparison of differences, and observe the degree of variation between different groups and within group samples. In Kyoto Encyclopedia of Genes and Genomes (KEGG) database search for the classification and pathway information of identified compounds in HMDB and lipidmaps databases. According to the grouping information, calculate and compare the multiple of differences, and use t-test to calculate the significance p-value of the differences in each compound. OPLS-DA modeling was performed using the R language package ropls, and 200 permutation tests were conducted to verify the reliability of the model. The VIP value of the model is calculated using multiple cross validation. Using the OPLS-DA model for differential multiples. The method of combining p-value and VIP value is used to screen differential metabolites. The screening criteria are FC>1 and p-value<0.05, VIP > 1. Use hypergeometric distribution test to calculate the significance values of differential metabolites in KEGG pathway enrichment analysis.

## 3 Results

### 3.1 *Apocynum pictum* has better salt resistance than *Apocynum venetum* during the seed germination stage

**Figure 1:**
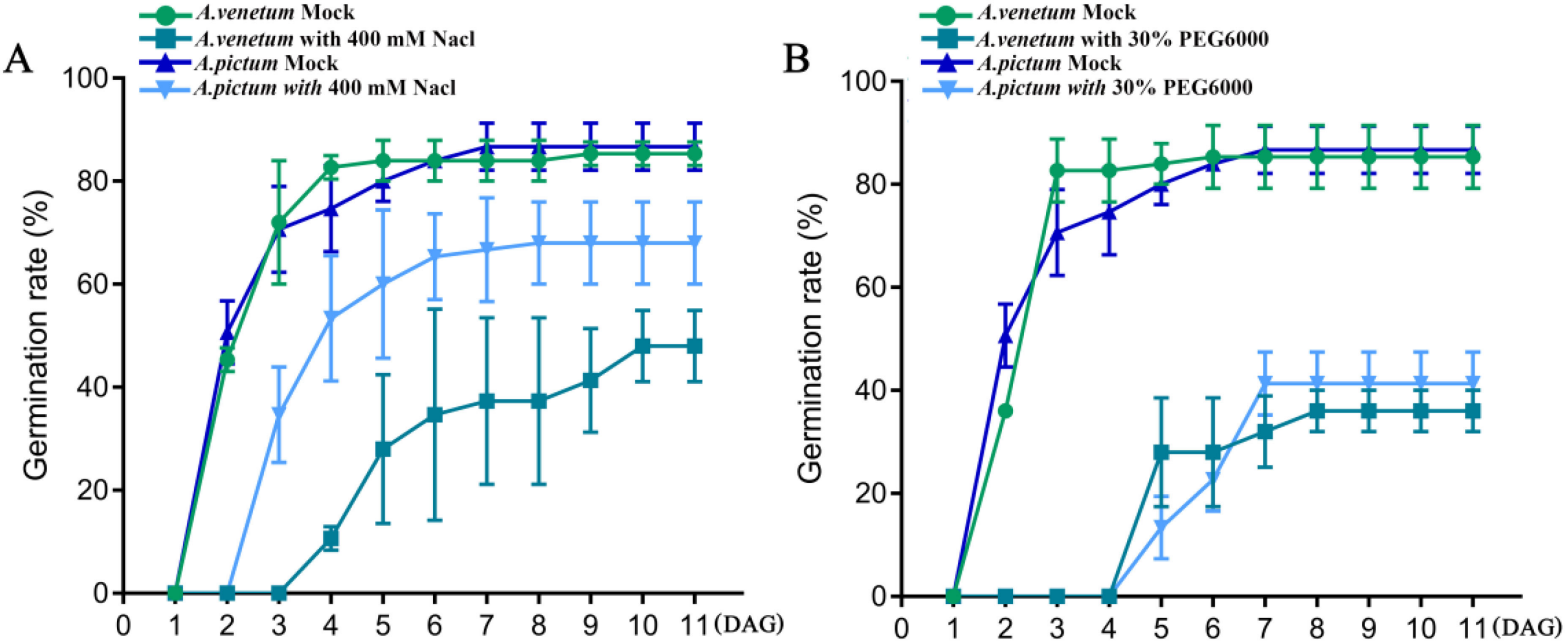
The effects of salt stress **(A)** and drought stress **(B)** on seed germination of *A. pictum* and *A. venetum*. The seeds of *A. pictum* and *A. venetum* were germinated on 1/2 MS solid medium containing 0 mM NaCl and 400 mM NaCl, respectively, and the germination rates of the seeds were measured after different germination days. Similarly, germination rate was measured on a medium containing 30% PEG6000. DAG, days after germination. The value is mean ± standard deviation (mean ± SD). Each experiment was repeated 3 times (n=3).

*Apocynum* can grow on land with high levels of salinization and drought, so we first investigated the differences in salt and drought resistance between two types of *Apocynum* seeds. We first screened the most appropriate NaCl concentration for salt stress treatment on *A. pictum* and *A. venetum* seed germination. We set NaCl at 0 mM (control group, CK), 50 mM, 100 mM, 200 mM, 400 mM, and 600 mM to treat *A. pictum* and *A. venetum* seeds during germination. The results showed that both of them could not germinate normally at a concentration of 600 mM Nacl (**Supplementary Figure S1A**). Thus, we set the final measured concentration of Nacl at 400 mM. There was no significant difference in germination rate between *A. pictum* and *A. venetum* seeds without any salt stress treatment. However, under 400 mM NaCl treatment, the germination time of both seeds was delayed compared to the control. The germination rate of *A. pictum* is significantly higher than that of *A. venetum*, and also, the seeds of *A. pictum* germinated one day earlier than *A. venetum* (**Figure 1A**). This implied that the superior salt tolerance of *A. pictum* seeds arises from earlier germination onset and higher germination rates under salt stress.

Subsequent drought resistance evaluation of both seed types was conducted following determination of optimal PEG6000 concentration for drought simulation. Initial screening revealed complete germination inhibition at 40% PEG6000 concentration, establishing 30% as the experimental drought stress condition (**Supplementary Figure S1B**). Comparative analysis revealed comparable germination kinetics between *A. pictum* and *A. venetum* under both control and drought conditions. While drought stress induced a consistent 3-day germination delay in both species relative to controls, final germination rates remained statistically indistinguishable between the two species (**Figure 1B**). These findings demonstrate that although *A. pictum* and *A. venetum* seeds exhibit differential salt tolerance, they maintain equivalent drought resistance capabilities under tested conditions.

### 3.2 ICP-MS analysis of the differences in elemental content between *A. pictum* and *A. venetum*

The intrinsic elemental composition of seeds serves as critical determinants of germination efficiency and subsequent seedling establishment. To investigate whether interspecific variations in seed elemental profiles between *A. pictum* and *A. venetum* contribute to differential salt tolerance capacities, we conducted a systematic comparative analysis of 17 essential mineral elements through ICP-MS.

**Figure 2:**
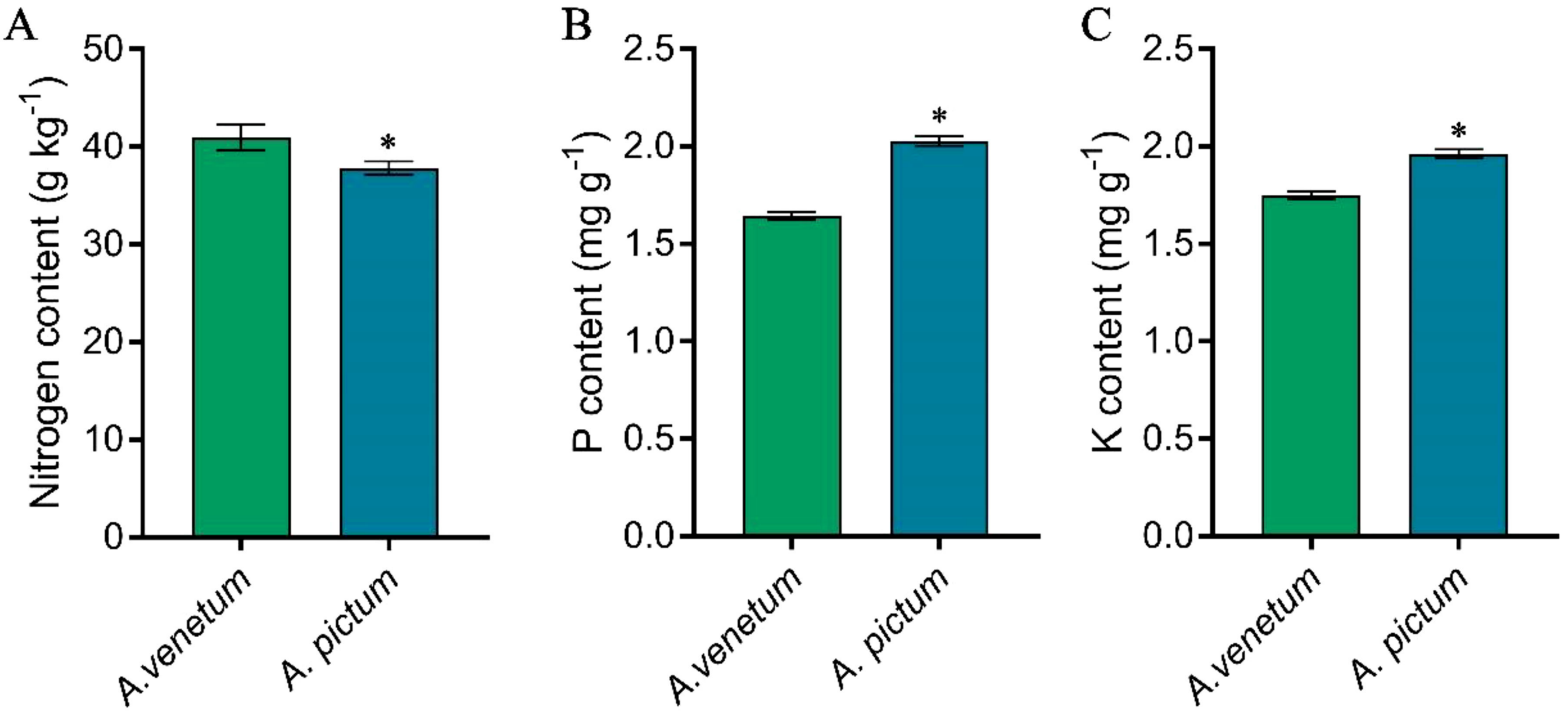
Determination of nitrogen **(A)**, phosphorus **(B)**, and potassium **(C)** content in *A. pictum* and *A. venetum* seeds. The content of different elements in *A. pictum* and *A. venetum* seeds was determined by ICP-MS after microwave digestion. The value is mean ± standard deviation (mean ± SD). Each experiment was repeated 3 times (n=3). * indicated there is a significant difference (P<0.05) between the representative values.

In terms of abundant elements, including nitrogen, phosphorus, and potassium, the results showed that *A. venetum* had significantly higher nitrogen content than *A. pictum*; However, the content of phosphorus and potassium in seeds of *A. venetum* is significantly lower than that in *A. pictum* seeds (**Figure 2**).

**Figure 3:**
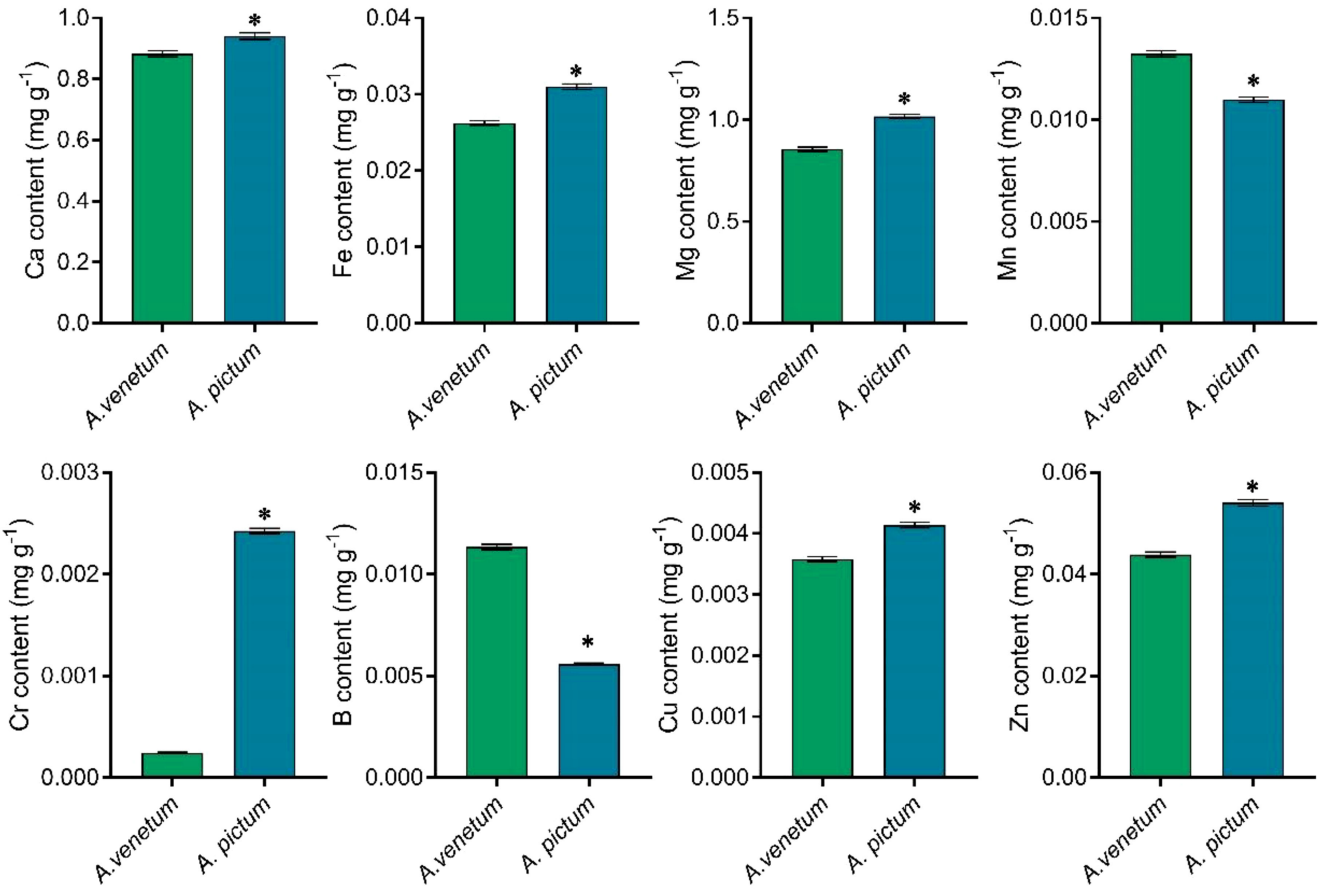
Determination of trace element content with high content in *A. pictum* and *A. venetum* seeds. The contents of various elements including calcium (Ca), iron (Fe), magnesium (Mg), manganese (Mn), chromium (Cr), boron (B), copper (Cu), and zinc (Zn) were determined by ICP-MS. The value is mean ± standard deviation (mean ± SD). Each experiment was repeated 3 times (n=3). * indicated there is a significant difference (P<0.05) between the representative values.

In addition to quantifying nitrogen, phosphorus, and potassium concentrations, we performed elemental analysis on both seed types. Comparative analysis revealed distinct differences in trace element profiles between *A. pictum* and *A. venetum* seeds. Notably, *A. pictum* seeds exhibited significantly lower boron (B) and manganese (Mn) concentrations compared to their *A. venetum* counterparts. Conversely, we observed substantially higher accumulation of calcium (Ca), iron (Fe), magnesium (Mg), chromium (Cr), copper (Cu), and zinc (Zn) in *A. pictum* seeds relative to *A. venetum* seeds (**Figure 3**). The most remarkable disparity was observed in chromium levels, with *A. pictum* seeds containing 8.88 times more Cr than *A. venetum* seeds.

**Figure 4:**
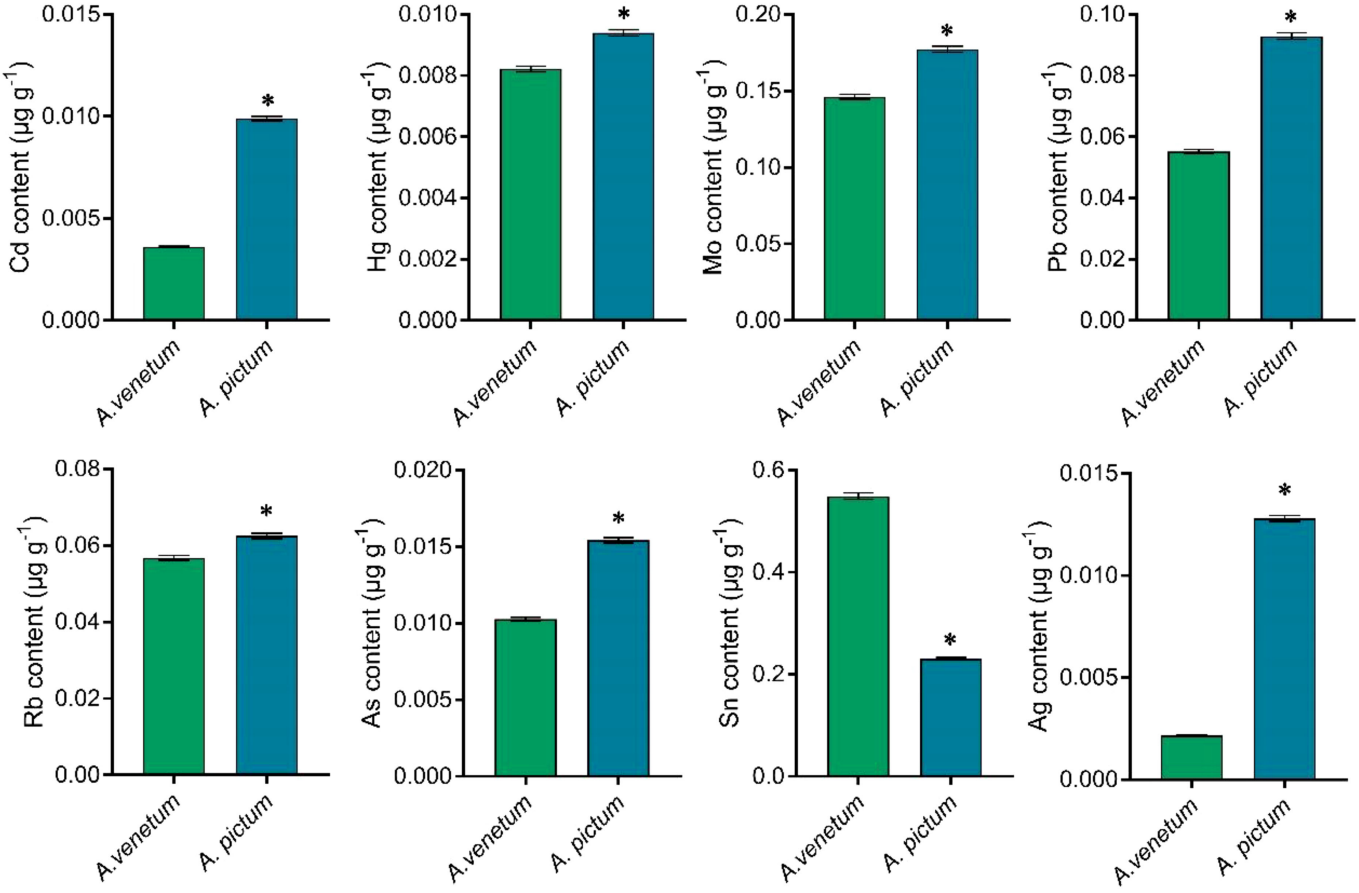
Determination of trace element content with lower content in *A. pictum* and *A. venetum* seeds. The contents of various elements including cadmium (Cd), hydrargyrum (Hg), molybdenum (Mo), lead (Pb), rubidium (Rb), arsenic (As), tin (Sn), and silver (Ag) were determined by ICP-MS. The value is mean ± standard deviation (mean ± SD). Each experiment was repeated 3 times (n=3). * indicated there is a significant difference (P<0.05) between the representative values.

Divergent patterns were observed in trace element composition between the two species. While *A. pictum* seeds exhibited a substantial 66.67% reduction in tin (Sn) content compared to *A. venetum* seeds, they demonstrated significantly elevated concentrations of other analyzed elements (**Figure 4**).

### 3.3 Detection of metabolite types and contents in *A. pictum* and *A. venetum* seeds using widely targeted metabolomics

Beyond elemental composition disparities contributing to post-germination physiological variations in seeds and seedlings, the differential accumulation of seed metabolites plays an equally crucial regulatory role. To elucidate the metabolic basis underlying these phenotypic variations, we conducted comprehensive metabolomic profiling of *A. pictum* (Baima BM) and *A. venetum* (Hongma HM) seeds using widely targeted metabolomics. This approach enabled systematic identification and comparative analysis of both primary and secondary metabolites across the two *Apocynum* species.

**Figure 5:**
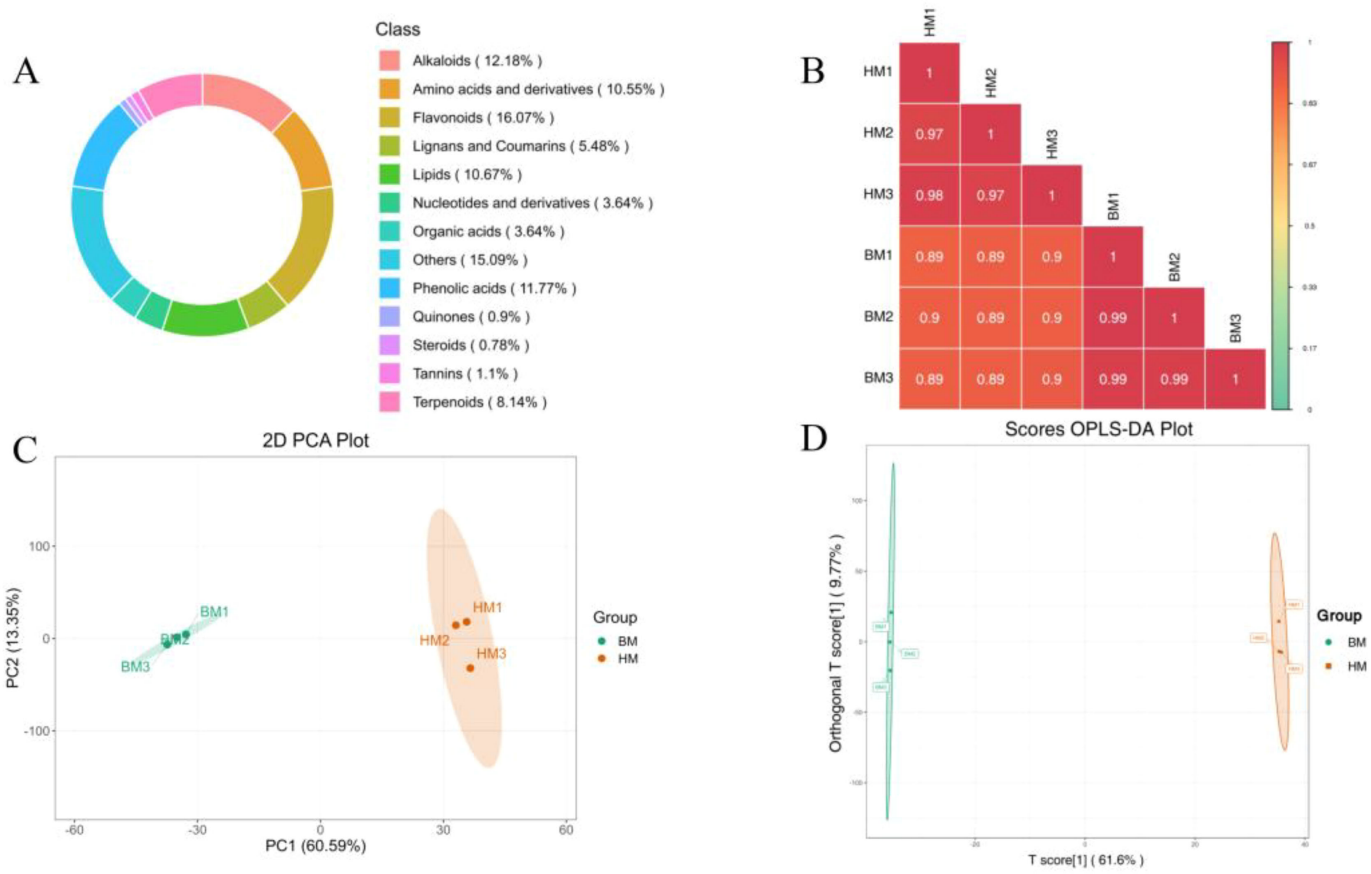
Data quality assessment of widely targeted metabolomics on *A. pictum* (BM) and *A. venetum* (HM) seeds. **(A)** Circular chart of metabolite categories. Each color represents a metabolite category, and the area of the color block indicates the proportion of that category; **(B)** Correlation diagram between samples. The vertical and diagonal lines respectively represent the sample names of different samples, and different colors represent different the pearson correlation. The redder the color, the stronger the positive correlation; And the greener the color, the weaker the correlation. The correlation coefficient between the two samples is marked in the square; **(C)** 2D principal component analysis (PCA) plot. PC1 represents the first principal component, PC2 represents the second one, and percentages represent the explanatory power of the principal component on the dataset; Each point in the figure represents a sample, samples in the same group are represented by the same color; **(D)** Scores OPLS-DA plot. The horizontal axis represents the predicted principal components, and the difference between groups can be seen in the direction of the horizontal axis; The vertical axis represents the orthogonal principal components, and the difference within the group can be seen in the direction of the vertical axis; The percentage represents the explanatory power of the component on the dataset. Each point in the figure represents a sample, samples in the same group are represented by the same color.

Metabolite profiling was performed through mass spectrometry-based qualitative and quantitative analyses using a local metabolic database. Our investigation identified 2,446 distinct metabolites, comprising: 258 amino acids and derivatives (10.5%), 89 nucleotides and derivatives (3.6%), 288 phenolic acids (11.8%), 89 organic acids (3.6%), 261 lipids (10.7%), 393 flavonoids (16.1%), 298 alkaloids (12.2%), 199 terpenes (8.1%), and 571 unclassified compounds (23.4%) (**Supplementary Table S1**). As illustrated in **Figure 5A**, flavonoids emerged as the predominant metabolite class, followed sequentially by alkaloids and phenolic acids. This compositional pattern aligns with previous metabolic characterizations of *Apocynum* foliar tissues, suggesting potential metabolic inheritance from maternal seed reserves to developing seedlings.

By conducting principal component analysis (PCA) on samples (including quality control samples), we can gain a preliminary understanding of the overall metabolite differences between each group of samples and the magnitude of variability among samples within the groups. The PCA results showed the trend of metabolic group separation between groups, indicating whether there are differences in metabolic groups within the sample group. The results showed that the inter group correlation coefficients of different samples were all greater than 0.85, indicating high experimental reproducibility (**Figure 5B**). We conducted PCA (**Figure 5C**) and orthogonal partial least squares-discriminant analysis (OPLS-DA) (**Figure 5D**) on metabolites in *A. pictum* and *A. venetum* seeds. OPLS-DA analysis is a multivariate statistical method with supervised recognition patterns that can effectively eliminate irrelevant influences and screen for differential metabolites. In this model, R2X and R2Y represent the explanatory power of the constructed model on the X and Y matrices, respectively. Q2 represents the predictive ability of the model. A Q2 higher than 0.95 in the comparison group indicates that the constructed model is appropriate (**Figure 5D**). The results showed that overall, there was no significant difference in the main components of metabolites within each group of *Apocynum* seeds, but there were significant differences between the two groups.

### 3.4 Further screening and identification of differential metabolites in *A. pictum* and *A. venetum* seeds

**Figure 6:**
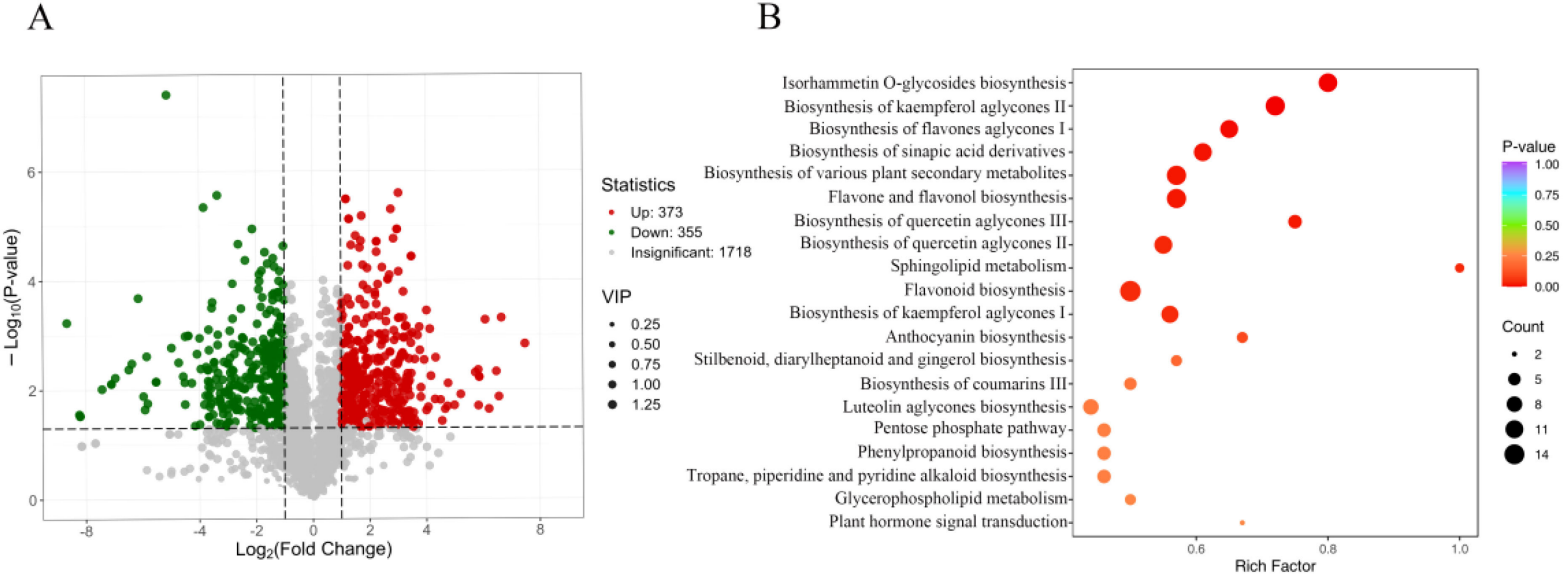
Screening and identification of differential metabolites in seeds of *A. pictum* and *A. venetum*. **(A)** Volcanic diagram of differential metabolites. Each point in the volcano diagram represents a metabolite, where green points represent down regulated differential metabolites, red points represent up regulated differential metabolites, and gray points represent detected metabolites with insignificant differences; The horizontal axis represents the logarithmic value (log_2_FC) of the relative content difference multiple of a metabolite between two groups of samples. The larger the absolute value of the horizontal axis, the greater the relative content difference of the substance between the two groups of samples. Under the VIP+FC+P-value screening conditions: the y-axis represents the level of significant difference (-log_10_ P-value), and the size of the dots represents the VIP value. Under the VIP+FC screening conditions: the vertical axis represents the VIP value, and the larger the vertical axis value, the more significant the difference, indicating that the screened differential metabolites are more reliable. **(B)** Enrichment map of differential metabolite pathways. The horizontal axis represents the RichFactor corresponding to each pathway, and the vertical axis represents the pathway name (sorted by P-value). The color of the dots reflects the P-value size, and the redder the dots, the more significant the enrichment. The size of the dots represents the number of enriched differential metabolites.

By applying Variable Importance in Projection (VIP) and Fold Change (FC) criteria to screen differential metabolites, those with VIP>1 and FC ≥ 2 or FC ≤ 0.5 were identified as significant. Comparative analysis of seed metabolites between *A. pictum* and *A. venetum* revealed 865 differential metabolites under these parameters, comprising 436 downregulated and 429 upregulated metabolites in *A. venetum* relative to *A. pictum* (**Supplementary Table S1**). When incorporating additional statistical significance through combined VIP, FC, and P-value criteria (VIP+FC+P-value), 728 differential metabolites were detected. The volcano plot (**Figure 6A**) illustrates this refined analysis, showing 355 metabolites downregulated and 373 upregulated in *A. venetum* compared to *A. pictum*.

To systematically categorize the differential metabolites across comparison groups, all identified compounds were cross-referenced with the KEGG database for pathway annotation. Subsequent enrichment analysis of the annotated metabolites was conducted to identify pathways exhibiting significant metabolite enrichment (**Figure 6B**). The twenty most significantly enriched metabolic pathways revealed that differentially abundant metabolites between *A. venetum* (HM) and *A. pictum* (BM) were predominantly associated with primary metabolic processes, including amino acid and carbohydrate metabolism pathways. Notably, our analysis also identified enrichment in secondary metabolic pathways, particularly those involving flavonoid biosynthesis, organic acid metabolism, and alkaloid synthesis pathways (**Figure 6B**).

### 3.5 Differential analysis of main primary metabolites in *A. pictum* and *A. venetum* seeds

We conducted a comparative analysis of primary metabolites between the two *Apocynum* seed varieties. Our investigation revealed 93 carbohydrate compounds exhibiting differential accumulation. Notably, *A. venetum* seeds demonstrated a marked 1.76-fold elevation in D-ribose content compared to *A. pictum*. Conversely, 25 carbohydrate metabolites in *A. venetum* seeds exhibited significantly lower levels than those in *A. pictum*, with Octyl-beta-D-glucopyranoside showing the most pronounced disparity (3.01-fold higher concentration in *A. pictum*, **Table 1**). The remaining 67 carbohydrate compounds (67/93, 72.04%) showed comparable accumulation patterns between both varieties, with no statistically significant differences observed (**Supplementary Table S2**).

**Table 1:** Changes in the content of sugar compounds in seeds of *A. pictum* (BM) and *A. venetum* (HM).

Analytes			BM1	BM2	BM3	HM1	HM2	HM3	VIP	P-value	FDR	Fold_Change	Log2FC	Type
Database Number	Metabolite Name	Formula												
Zmgn000173	D-Ribose	C5H10O5	111266.95	111214.66	125659.46	23133.511	27025.021	52969.75	1.181	0.0045	0.028	3.37580606	1.7552	up
MWSmce220	D-Glucono-1,5-lactone	C6H10O6	18629.038	17847.176	31705.984	50442.128	42459.867	45259.807	1.131	0.0191	0.06	0.49349528	-1.019	down
pme2253	L-Gulono-1,4-lactone	C6H10O6	18629.038	17847.176	31705.984	50442.128	42459.867	45259.807	1.131	0.0191	0.06	0.49349528	-1.019	down
pme0534	D-Gluconic acid	C6H12O7	1703874.6	1988747.8	1968262.2	3452546.5	3721736.5	4302368.7	1.242	0.0094	0.04	0.49325227	-1.02	down
Lmxn000380	Digalactosylglycerol	C15H28O13	96923.917	105738.52	109933.96	203063.9	227641.35	217941.57	1.262	0.0007	0.013	0.48192081	-1.053	down
MWSslk235	Allitol	C6H14O6	58455.974	82332.392	66600.539	142137.78	144902.72	148414.1	1.231	0.0057	0.031	0.47625839	-1.07	down
Wafn005903	2,4-DiSucrose	C22H38O13	24040.394	13903.783	26716.24	36672.036	39481.213	60251.057	1.061	0.0646	0.127	0.47403501	-1.077	down
Zmpn000199	D-Galactarate	C6H10O8	662672.13	604649.47	615898.49	1220149.5	1355963.4	1480475.7	1.258	0.0079	0.036	0.46423738	-1.107	down
Zmyn000108	D-Glucarate	C6H10O8	654330.83	617876.84	593584.71	1348622	1380088.1	1442020.2	1.27	7E-05	0.004	0.44735388	-1.161	down
MWSmce486	D-Gal alpha 1->6D-Gal alpha 1->6D-Glucose	C18H32O16	131242.61	126724	105971.97	300377.13	239358.76	286521.32	1.241	0.0067	0.033	0.44046646	-1.183	down
Wcsn000472	Laminarin	C18H32O16	1205250.3	1257761.9	1174171.9	2827033.1	2667693.9	2789978.7	1.272	0.0001	0.006	0.43902394	-1.188	down
pmb2653	DMelezitose O-rhamnoside	C24H42O20	381537.72	364094.16	307157.4	709097.81	965026.87	808529.69	1.233	0.0169	0.056	0.42405793	-1.238	down
pme0500	Melezitose	C18H32O16	807475.1	767904.22	802732.98	1974543	2022456.9	2088794.9	1.273	0.0002	0.007	0.39076446	-1.356	down
mws4163	Nystose	C24H42O21	187593.34	167446.66	164087.24	439743.92	483307.75	418425.94	1.264	0.0019	0.02	0.38698167	-1.37	down
pme2125	Raffinose	C18H32O16	1719470.4	1707759.9	1382389.4	4527517.1	4797610.4	3641984.5	1.243	0.0104	0.041	0.37090909	-1.431	down
mws1589	Panose	C18H32O16	1719470.4	1707759.9	1382389.4	4527517.1	4797610.4	3641984.5	1.243	0.0104	0.041	0.37090909	-1.431	down
mws1155	D-Mannitol	C6H14O6	16724.525	16169.341	13910.509	41748.792	42587.542	48875.374	1.262	0.0025	0.023	0.3513533	-1.509	down
pme2237	Galactitol	C6H14O6	16724.525	16169.341	13910.509	41748.792	42587.542	48875.374	1.262	0.0025	0.023	0.3513533	-1.509	down
Zbkn000353	myo-Inositol 5-phosphate	C6H13O9P	383065.12	322267.99	371116.97	1120465.8	1010761.8	1187077	1.265	0.002	0.021	0.32439761	-1.624	down
mws1090	D-Glucose-1-phosphate	C6H13O9P	383065.12	322267.99	371116.97	1120465.8	1010761.8	1187077	1.265	0.002	0.021	0.32439761	-1.624	down
MWS2442	D-Fructose 6-Phosphate	C6H13O9P	383065.12	322267.99	371116.97	1120465.8	1010761.8	1187077	1.265	0.002	0.021	0.32439761	-1.624	down
mws0866	D-Glucose 6-phosphate	C6H13O9P	383065.12	322267.99	371116.97	1120465.8	1010761.8	1187077	1.265	0.002	0.021	0.32439761	-1.624	down
mws1593	Maltotetraose	C24H42O21	39209.259	32537.233	35219.733	104934.19	117406.71	128968.44	1.263	0.0045	0.028	0.30447874	-1.716	down
Lmqn000213	Stachyose	C24H42O21	77971	97057.616	84933.583	256958.62	339850.12	310084.94	1.257	0.0095	0.04	0.28665124	-1.803	down
Safn000636	6-O-D-glycero-α-D-manno-heptopyranosyl-β-D-glucopyranose	C13H24O12	36527.995	61009.021	43950.009	151974.85	163232.85	263335.18	1.212	0.0492	0.107	0.24455754	-2.032	down
Lcsn007077	Octyl-Beta-D-Glucopyranoside	C14H28O6	14039.656	14600.945	14526.517	99891.459	146737.26	101110.55	1.264	0.0223	0.065	0.12413645	-3.01	down

The ones with the most significant decreases in HM are placed on the far left of the graph, while the ones with the most significant increases are placed on the far right of the graph. The X axis represents the code of each compound, and the specific compound name referred to can be found in the attached table. Asterisks denote statistically significant differences (* p < 0.05).

**Figure 7:**
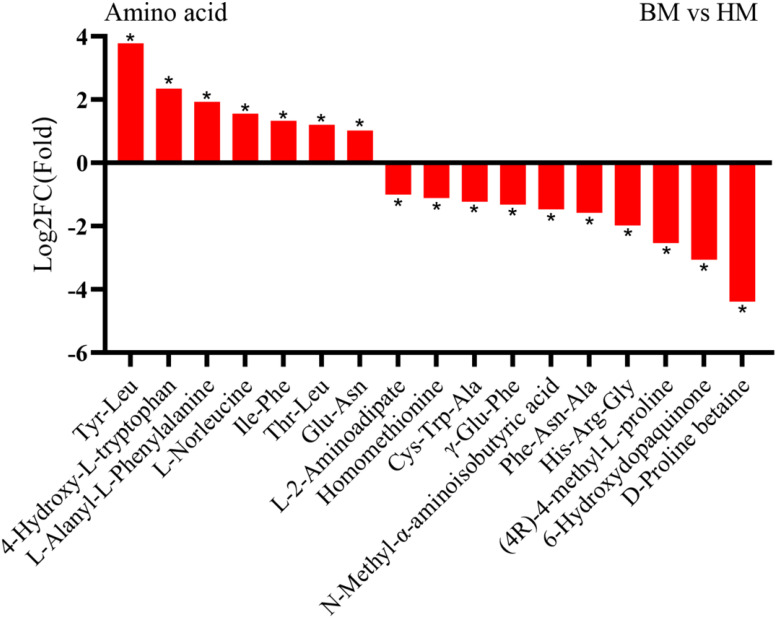
Changes in the content of amino acid compounds in seeds of *A. pictum* (BM) and *A. venetum* (HM). The ones with the most significant decreases in HM are placed on the far left of the graph, while the ones with the most significant increases are placed on the far right of the graph. The X axis represents the code of each compound, and the specific compound name referred to can be found in the attached table. Asterisks denote statistically significant differences (* p < 0.05).

In addition to sugar compounds, our analysis identified 258 amino acid compounds exhibiting diverse content levels. Subsequent screening demonstrated that *A. venetum* seeds contained 28 amino acid compounds at markedly elevated concentrations compared to *A. pictum* seeds. Notably, the most pronounced content disparities were observed in Tyr-Leu and Ile-Trp, showing 3.78-fold and 3.54-fold increases, respectively (**Figure 7**). Conversely, comparative analysis revealed 46 amino acid compounds with notably higher abundance in *A. pictum* seeds relative to *A. venetum* seeds (**Supplementary Table S3**).

### 3.6 Differential analysis of flavonoid metabolites in *A. pictum* and *A. venetum* seeds

**Figure 8:**
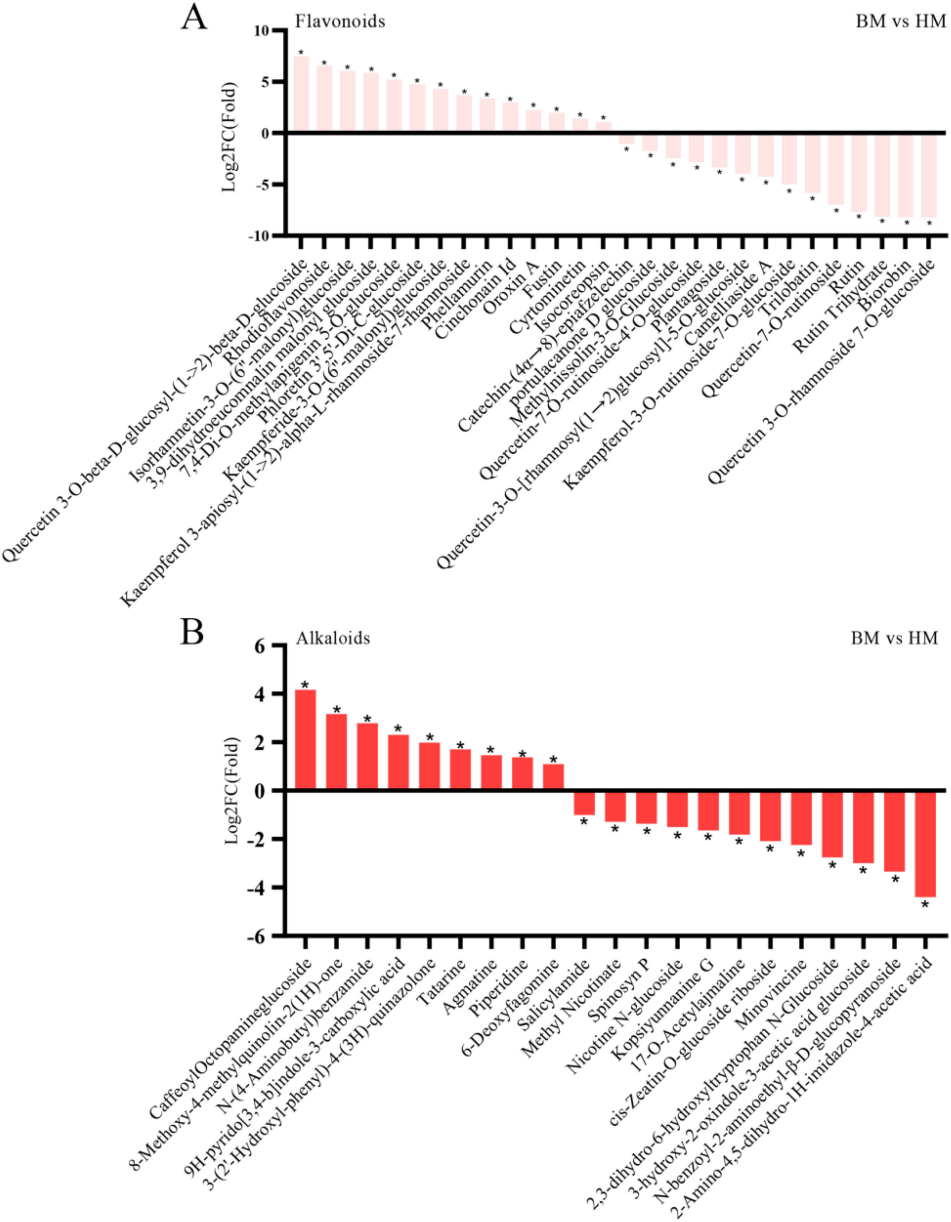
Changes in the content of flavonoids **(A)** and alkaloids **(B)** compounds in seeds of *A. pictum* (BM) and *A. venetum* (HM). The ones with the most significant decreases in HM are placed on the far left of the graph, while the ones with the most significant increases are placed on the far right of the graph. The X axis represents the code of each compound, and the specific compound name referred to can be found in the attached table. Asterisks denote statistically significant differences (* p < 0.05).

In addition to primary metabolites, our investigation extended to secondary metabolites exhibiting differential accumulation between the two *Apocynum* seed types. Given the recognized status of *Apocynum* as a flavonoid-enriched species, we prioritized comparative analysis of flavonoid profiles. Our analysis identified 393 flavonoids showing significant inter-species variation, with 141 compounds demonstrating substantially higher concentrations in *A. venetum* seeds. Notably, the quercetin derivative 3-O-beta-D-glucosyl-(1->2)-beta-D-glucoside exhibited a remarkable 7.48-fold higher concentration in *A. venetum* compared to *A. pictum*. Conversely, 83 flavonoids displayed an inverse accumulation pattern, showing preferential accumulation in *A. pictum* seeds. Particularly striking was the 8.67-fold enrichment of phloretin-2’-O-(6’’-O-rhamnoside) glucoside in *A. pictum* relative to *A. venetum* (**Figure 8A**). Among the 90 quantified flavonols showing species-specific accumulation patterns, this subclass constituted the predominant proportion (**Supplementary Table S4**). These metabolic divergence patterns notably aligned with previous comparative analyses of leaf metabolites between *A. pictum* and *A. venetum*.

### 3.7 Differential analysis of organic acid and alkaloid metabolites in *A. pictum* and *A. venetum* seeds

We conducted a comparative analysis of organic acid and alkaloid profiles between the two species. Among 89 organic acids exhibiting differential abundance, only four compounds—jasmonic acid, asperic acid, 2-hydroxyethylphosphonate, and 2,2-dimethylsuccinic acid—were found at higher levels in *A. venetum* seeds compared to *A. pictum* seeds. Conversely, seven organic acids showed elevated concentrations in *A. pictum* seeds (**Table 2**). Notably, the maximum fold-changes observed in either direction did not exceed two-fold, suggesting relatively minor variations in organic acid composition between the two seed types (**Supplementary Table S5**).

**Table 2:** Changes in the content of organic acids compounds in seeds of *A. pictum* (BM) and *A. venetum* (HM).

Analytes			BM1	BM2	BM3	HM1	HM2	HM3	VIP	P-value	FDR	Fold_Change	Log2FC	Type
Database Number	Metabolite Name	Formula												
pme1654	(-)-Jasmonic acid	C12H18O3	188330.4	170883.6	276470.3	44766.49	49109	53913.9	1.24492	0.03732	0.089	4.301284687	2.1048	up
Lmhp007489	Asperic acid	C16H28O4	41888.37	74955.16	61847.87	15806.32	16257.37	26966.87	1.16595	0.03999	0.093	3.027099478	1.5979	up
Zmgn000216	2-Hydroxyethylphosphonate	C2H7O4P	193706.6	153553.1	183195.2	63717	74189.52	83656.67	1.23762	0.00525	0.03	2.394147317	1.2595	up
Wmzn000227	2,2-Dimethylsuccinic acid	C6H10O4	26145.15	36388	28335.48	11479.02	19252.32	12143.52	1.14162	0.01752	0.057	2.119392205	1.0837	up
pme2914	3-Hydroxy-3-methylglutarate	C6H10O5	773453.1	519152.2	849054.7	1549191	1548433	1390157	1.17977	0.00591	0.032	0.47722028	-1.0673	down
Wasn003197	3-(Beta-D-Glucopyranosyloxy)-5-Hydroxyhexanoic Acid Methyl Ester	C13H24O9	21895.95	39970.01	22844.19	69727.35	77712.76	47956.5	1.10566	0.03236	0.081	0.4335293	-1.2058	down
Wasyqn1008	Dimethylglyceric Acid Glucoside	C11H20O9	139438.5	89255.81	99317.65	272956.2	230735.6	268143.9	1.2103	0.00203	0.021	0.424976464	-1.2345	down
ML10176345	3-Dehydroshikimate	C7H8O5	26280.68	36686.63	21673.8	61842.16	60290.67	87139.9	1.17388	0.02424	0.068	0.404453621	-1.306	down
Rn04196	12-hydroxy jasmonoyl sulfate	C12H18O7S	18668325	20310924	19308225	60123237	60555753	66428148	1.27141	0.00141	0.017	0.311519244	-1.6826	down
mws0154	Shikimate	C7H10O5	34397.65	25960.32	27905.38	102278.6	97113.59	107718.3	1.26198	6.6E-05	0.004	0.287399373	-1.7989	down
ZbBn002068	3-methyl-ShikimicAcid	C8H12O5	6659.304	4147.048	10894.33	36562.35	33702.1	20341	1.1597	0.03103	0.078	0.23950747	-2.0619	down

The ones with the most significant decreases in HM are placed on the far left of the graph, while the ones with the most significant increases are placed on the far right of the graph. The X axis represents the code of each compound, and the specific compound name referred to can be found in the attached table. Asterisks denote statistically significant differences (* p < 0.05).

Our phytochemical analysis revealed a total of 298 identified alkaloids exhibiting distinct quantitative profiles between the two species. Comparative quantification demonstrated that *A. venetum* seeds contained significantly elevated concentrations (p<0.05) in 39 alkaloid compounds compared to *A. pictum* counterparts. Conversely, *A. pictum* seeds showed superior accumulation of 53 alkaloid constituents when contrasted with *A. venetum* samples, with these differential expression patterns being statistically validated through rigorous analytical methods (detailed in **Figure 8B** and supporting data in **Supplementary Table S6**). The remaining 206 alkaloids displayed comparable concentration levels between the two species within the detection thresholds of our analytical platform.

## 4 Discussion

Not only does *Apocynum* demonstrate exceptional adaptability to environments characterized by intense drought and high salinity (Xie et al., 2024), but it also serves as a valuable medicinal resource through its abundant synthesis of diverse secondary metabolites (Xie et al., 2012). This dual functionality underscores *Apocynum* significant potential for research and development applications.

**Figure 9:**
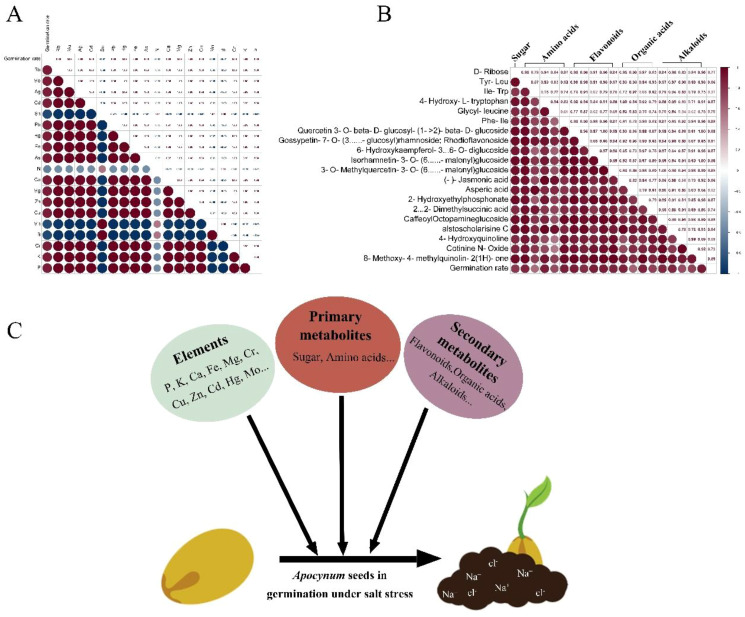
Correlation analysis between germination rate of *Apocynum* seeds under salt stress and element **(A)** and metabolite content **(B)**. The specific values of the Pearson correlation coefficient (from -1 to 1) are marked in the rectangle in the upper right corner. The bluer the color of the circle, the more negative the correlation, and the redder the color, the more positive the correlation. The size of the circle represents the magnitude of significance. **(C)** Schematic diagram of the possible mechanism of germination of *Apocynum* seeds under salt stress.

Mineral elements in seeds play critical roles in mediating stress responses during germination, particularly under saline conditions (Zhao et al., 2021a). Potassium (K^+^) functions as a key regulator of ionic homeostasis by counteracting sodium (Na^+^) accumulation, thereby maintaining cellular K^+^/Na^+^ ratios essential for normal physiological processes (Lata et al., 2022). Calcium (Ca²^+^) further enhances salt tolerance through dual mechanisms: stabilizing membrane integrity and modulating ion transporter activity via calcium-mediated signaling pathways (Seifikalhor et al., 2019; Qi et al., 2022; Mulaudzi et al., 2020; Rahman et al., 2016). In this study, comparative analysis of two *Apocynum* species revealed distinct salt tolerance capacities, with *A. pictum* seeds exhibiting significantly higher K^+^ and Ca²^+^ content than *A. venetum*, consistent with their superior germination performance under salt stress (Turhan and Kuscu, 2023). Under drought conditions, micronutrients such as copper (Cu²^+^) contribute to metabolic resilience through enzymatic activation, exemplified by its role as a cofactor in cytochrome oxidase for cellular respiration (Mansilla et al., 2019). Notably, while both species demonstrated comparable drought tolerance during germination, *A. pictum* exhibited significantly enhanced salt stress adaptation. Elemental profiling identified a positive correlation between seed germination rates under salt stress and the content of multiple minerals (K^+^, Ca²^+^, Cu²^+^, etc.), excluding nitrogen, manganese, and boron (**Figure 9A**). This suggests a coordinated mineral-mediated mechanism underlying salt tolerance in *Apocynum* seeds, where ionic regulation and metabolic maintenance jointly mitigate stress impacts.

Seed metabolites, encompassing both primary and secondary compounds, constitute fundamental biochemical determinants of stress resilience during early plant development. Soluble sugars serve dual roles as essential energy substrates and osmolytes, with their catabolic mobilization under saline stress enabling two critical adaptive responses: rapid energy provision for salt exclusion mechanisms, and osmotic adjustment through intracellular accumulation to counterbalance extracellular water potential gradients (Zhou et al., 2020; Abubakar et al., 2024; Merchant and Adams, 2005; Zhao et al., 2021b). This osmoregulatory function is particularly crucial during germination, where sugar-derived turgor maintenance prevents cellular dehydration damage in emerging radicles (Boriboonkaset et al., 2013). Amino acid metabolism similarly contributes to salinity adaptation, with proline emerging as a multifunctional protectant (Chen et al., 2020; Li et al., 2023; Rajkumari et al., 2023). Beyond its established role in osmotic equilibrium, proline stabilizes protein conformations through preferential exclusion effects—a biochemical safeguard against salt-induced macromolecular denaturation (Khedr et al., 2003; Vicente et al., 2004). Our comparative metabolomic analysis revealed elevated carbohydrate reserves in *A. pictum* seeds relative to *A. venetum*, a biochemical disparity that correlates with observed interspecific differences in salt-stressed germination efficiency. This finding aligns with established models where seed carbohydrate pools positively modulate ionic stress tolerance through sustained energy supply and cellular turgor maintenance (Hoque et al., 2007).

The enhanced salt tolerance of *A. pictum* seeds compared to *A. venetum* may be further attributed to their elevated secondary metabolite profiles, particularly flavonoids and alkaloids (Jiang et al., 2024; Khare et al., 2020; Abubakar et al., 2022). Flavonoids function as potent antioxidants to scavenge salt-induced reactive oxygen species (ROS), including superoxide anions and hydrogen peroxide, thereby alleviating oxidative damage to cellular components (Zhang et al., 2023; Gao et al., 2019). The dual contributions of antioxidant enzymes (e.g., SOD, POD, CAT) and secondary metabolites (e.g., flavonoids) to ROS scavenging in *A. pictum* seeds suggest a synergistic mechanism underpinning salt stress tolerance. Antioxidant enzymes function as the first line of defense by catalytically neutralizing ROS through sequential reactions—SOD converts superoxide radicals to hydrogen peroxide, which is subsequently detoxified by POD and CAT into water and oxygen (Zhao et al., 2021c). In contrast, flavonoids act as non-enzymatic antioxidants, directly quenching residual ROS (e.g., hydroxyl radicals) via electron donation and chelating metal ions that catalyze oxidative chain reactions (Speisky et al., 2022). This complementary interaction ensures comprehensive ROS elimination across different cellular compartments and stress phases. Alkaloids contribute to ionic homeostasis by chelating excessive Na^+^ ions, preventing their cytotoxic accumulation within cells (Dai et al., 2020; Yahyazadeh et al., 2018). This synergistic mechanism—combining oxidative stress mitigation and ion detoxification—likely underlies the observed interspecific differences in salt tolerance. Correlation analysis between seed metabolite content and germination rates under salt stress revealed significant positive associations for carbohydrates, amino acids, flavonoids, organic acids, and alkaloids. Notably, flavonoids exhibited the strongest correlation with germination performance, emphasizing their critical role in *Apocynum* seed adaptation to saline environments (**Figures 9B, C**). These findings collectively suggest that the superior salt tolerance of *A. pictum* seeds arises from the coordinated action of stress-protective metabolites, with secondary metabolites complementing primary metabolic adaptations.

The observed interspecific differences in elemental and metabolic profiles between *A. pictum* and *A. venetum* seeds raise a critical question regarding the origin of these traits: whether they are genetically determined or influenced by maternal environmental factors. In this study, seeds of both species were collected from the same geographic region (Yuli, Xinjiang) under comparable environmental conditions, minimizing potential maternal effects caused by divergent growth environments. This suggests that the higher accumulation of stress-protective elements (K^+^, Ca²^+^, etc.) and metabolites (flavonoids, alkaloids, etc.) in *A. pictum* seeds may primarily stem from genetic divergence between the two species. Previous genomic studies on *Apocynum* plants have found species-specific expression differences in gene families related to secondary metabolism (such as flavone 3-hydroxylase), which may be the basis for differential regulation of salt tolerance traits (Gao et al., 2023). However, we cannot entirely exclude the possibility of maternal environmental imprinting, as epigenetic modifications induced by abiotic stressors (e.g., salinity, drought) during seed development may transiently influence metabolic profiles (Rajkumari et al., 2023). Future studies involving reciprocal transplantation experiments or controlled maternal environment trials would help disentangle genetic and environmental contributions to these traits.

## 5 Conclusion

Our research has demonstrated significant differences in the responses of Apocynum pictum and A. venetum to salt stress during seed germination, while no notable differences were observed between these two species regarding drought stress. Utilizing inductively coupled plasma mass spectrometry (ICP-MS) alongside comprehensive targeted metabolomics analysis, we discovered that Apocynum pictum seeds exhibit a greater tolerance to salt stress during germination. This enhanced tolerance may be attributed to elevated concentrations of essential elements such as potassium ions (K^+^), calcium ions (Ca^2+^), and copper ions (Cu^2+^) present in their seeds. Furthermore, Apocynum pictum possesses a more diverse array of primary and secondary metabolites, the synergistic effects of these metabolites and ions are likely pivotal to its superior salt tolerance.

## Data Availability

The original contributions presented in the study are included in the article/**Supplementary Material**. Further inquiries can be directed to the corresponding authors.
