# Supplementary material for: Comprehensive analysis of element and metabolite content between the seeds of Apocynum venetum and Apocynum pictum provides new sights for the salt tolerance in Apocynum
Source: Front Plant Sci. 2025 Jun 25;16:1611975. doi: 10.3389/fpls.2025.1611975 (PMC12239748; doi:10.3389/fpls.2025.1611975)
Supplement: Supplementary file 1 [file Table1.docx]

**Supplementrary Figure**


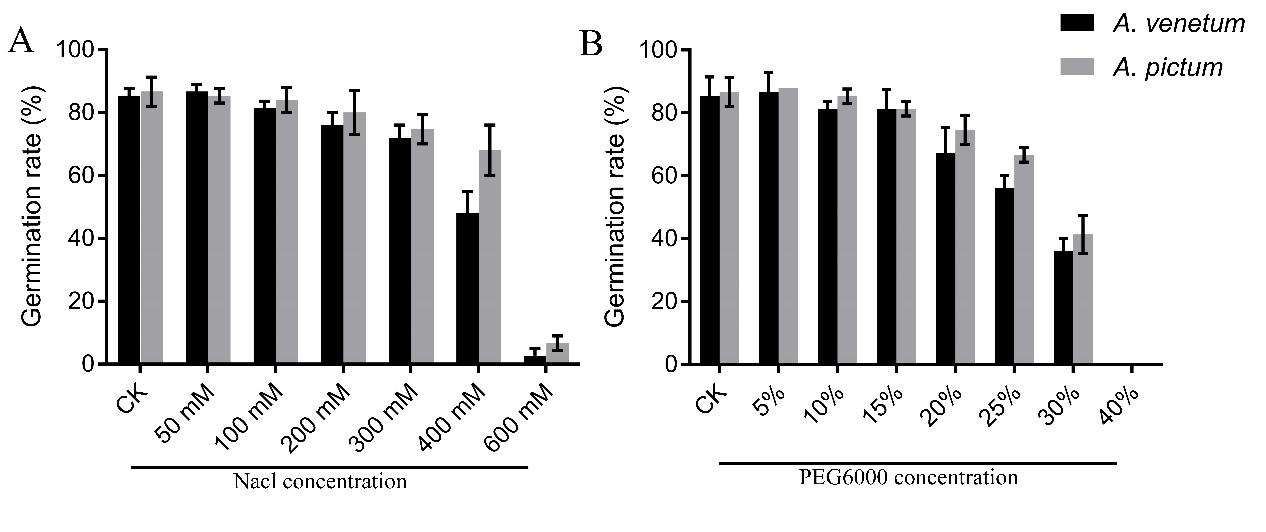


**Figure S1.** Seed germination rates of *A. pictum* and *A. venetum* under salt stress (A) and drought stress (B). The value is mean±standard deviation (mean±SD). Each experiment was repeated 3 times (n=3).
